# Association of the Common Genetic Polymorphisms and Haplotypes of the Chymase Gene with Left Ventricular Mass in Male Patients with Symptomatic Aortic Stenosis

**DOI:** 10.1371/journal.pone.0096306

**Published:** 2014-05-13

**Authors:** Ewa Orlowska-Baranowska, Jaroslaw Gora, Rafal Baranowski, Patrycjusz Stoklosa, Lucja Gadomska vel Betka, Ewa Pedzich-Placha, Malogrzata Milkowska, Marta K. Koblowska, Tomasz Hryniewiecki, Zbigniew Gaciong, Grzegorz Placha

**Affiliations:** 1 Department of Valvular Heart Diseases, Institute of Cardiology, Warsaw, Poland; 2 Department of Internal Medicine, Hypertension, and Vascular Diseases, Medical University of Warsaw, Warsaw, Poland; 3 Clinic of Arrhythmia, Institute of Cardiology, Warsaw, Poland; 4 Faculty of Biology, University of Warsaw, Warsaw, Poland; 5 Institute of Biochemistry and Biophysics, Polish Academy of Sciences, Warsaw, Poland; Medical University Innsbruck, Austria

## Abstract

We investigated the association between polymorphisms and haplotypes of the chymase 1 gene (*CMA1*) and the left ventricular mass index (LVM/BSA) in a large cohort of patients with aortic stenosis (AS). Additionally, the gender differences in cardiac remodeling and hypertrophy were analyzed. The genetic background may affect the myocardial response to pressure overload. In human cardiac tissue, CMA1 is involved in angiotensin II production and TGF-β activation, which are two major players in the pathogenesis of hypertrophy and fibrosis. Preoperative echocardiographic data from 648 patients with significant symptomatic AS were used. The LVM/BSA was significantly lower (p<0.0001), but relative wall thickness (RWT) was significantly higher (p = 0.0009) in the women compared with the men. The haplotypes were reconstructed using six genotyped polymorphisms: rs5248, rs4519248, rs1956932, rs17184822, rs1956923, and rs1800875. The haplotype h1.ACAGGA was associated with higher LVM/BSA (p = 9.84×10^−5^), and the haplotype h2.ATAGAG was associated with lower LVM/BSA (p = 0.0061) in men, and no significant differences were found in women. Two polymorphisms within the promoter region of the *CMA1* gene, namely rs1800875 (p = 0.0067) and rs1956923 (p = 0.0015), influenced the value of the LVM/BSA in males. The polymorphisms and haplotypes of the *CMA1* locus are associated with cardiac hypertrophy in male patients with symptomatic AS. Appropriate methods for the indexation of heart dimensions revealed substantial sex-related differences in the myocardial response to pressure overload.

## Introduction

Left ventricular hypertrophy (LVH), which develops due to pressure overload in patients with aortic stenosis [1], is associated with increased mortality and morbidity before and after aortic valve surgery [2]. The lack of an evident correlation between the stenosis-dependent pressure load and the degree of LVH [3] suggests that the left ventricular phenotype is dependent on a polygenic background [4]. The familial predisposition of LVH is supported by several studies [5], [6].

Angiotensin II is known to promote hypertrophy of cardiac myocytes and hyperplasia of cardiac fibroblasts [7]. Angiotensin II is generated locally in the cardiac tissue independently of circulating angiotensin II [8], and chymase has been found to be the major angiotensin II–producing enzyme in the human heart [9]. In response to pathological conditions, such as pressure or volume overload, myocardial infarction, and diabetes [8], the locally generated angiotensin II contributes to the development of cardiac hypertrophy and fibrosis [10].

Chymase is also involved in the activation of TGF-β in endothelial cells and in the heart [10]. It has been shown that TGF-β plays an important role in the pathogenesis of fibrotic and hypertrophic remodeling in the pressure-overloaded heart, both in animal models and in patients with aortic stenosis [11].

The expression of the chymase gene and its enzymatic activity in cardiac tissue was found to gradually increase over time in a hamster model of pressure overload hypertrophy [12], and several studies have suggested the role of chymase in left ventricular remodeling [10].

The *CMA1* gene is located on the long arm of chromosome 14, and its polymorphisms are associated with asthma (STR and (TG)_n_(GA)_m_ repeat) [13], 14, atopic eczema (rs1800875) [15], heart failure of nonischemic etiology, and low ejection fraction (rs1800875) [16]. However, no functional polymorphism has yet been reported.

The goal of this study was to examine the association between genetic variations in the *CMA1* locus and LVH using a comprehensive tagging polymorphism and haplotype approach in a large cohort of patients with aortic stenosis.

## Materials and Methods

### Patients

The study group comprised patients treated in the Department of Valvular Heart Diseases at the Institute of Cardiology in Warsaw who were referred for surgical intervention due to significant aortic stenosis. Patients with coexisting significant mitral and/or tricuspid disease or moderate/severe aortic regurgitation were excluded from the study. Written informed consent to participate was obtained from all of the patients, and the study was approved by the Ethics Committee of the Medical University of Warsaw and the Institute of Cardiology. All of the patients with a history of angina or aged >50 years underwent coronary arteriography. Significant coronary artery disease was defined as a reduction of at least 70% in the diameter of a major coronary artery or a 50% reduction in the left main coronary artery diameter.

### Echocardiography

The standardized examination comprised two-dimensional guided M-mode transthoracic echocardiograms and selected two-dimensional Doppler recordings. The left ventricular mass (LVM) was calculated according to the Penn Convention modified by Devereux LVM (g) = 1.04[(IVST+LVEDD+PWT)^3^ − LVEDD^3^] −13.6 [17] where LVEDD is the left ventricular end-diastolic diameter, IVST is the intraventricular septal thickness in diastole, and PWT is the posterior wall thickness in diastole. The body surface area (BSA) was calculated using the modified DuBois formula: BSA = (height (m)^0.73^ × body weight (kg)^0.4^ × 71.84)/10,000 [18]. The relative wall thickness (RWT) was calculated as RWT = PWT/(0.5) LVEDD. The left ventricular geometry was classified as previously described [1]: a) concentric hypertrophy, increased LVM/height and increased RWT; b) eccentric hypertrophy, increased LVM/height and normal RWT; c) concentric remodeling, normal LVM/height and increased RWT; and d) normal geometry, normal LVM/height and normal RWT. An LVM/height of 143 g/m or less for men and of 102 g/m or less for women and an RWT equal to at most 0.45 were considered normal.

### Polymorphism Selection

The single nucleotide polymorphisms (SNPs) were selected from the HapMap database (www.hapmap.org) to capture the genetic diversity of the region from 2.6 kb upstream to 5.1 kb downstream of the *CMA1* gene in a population of Utah residents with Northern and Western European ancestry. Haplotype blocks were defined using the method described by Gabriel et al. [19]. The haplotype tagging SNPs (htSNPs) were selected using the method described by Stram et al. [20] to predict the common haplotypes among Caucasians that meet a criterion of R_h_^2^>0.80.

### Genotyping and Sequencing

The SNP genotyping was performed using the SNPlex Genotyping system or TaqMan technology (Applied Biosystems, Foster City, CA, USA). The SNPlex assay was designed by the manufacturer, and the experiments were conducted according to the standard protocol. The analysis of the SNPlex products was performed using the ABI3730xl genetic analyzer and the GeneMapper 4.0 software. Predesigned TaqMan assays were obtained from the manufacturer, and the genotyping was performed using a standard protocol on a ViiA7 Real-Time PCR System. Ten percent of the samples were analyzed duplicates for quality control. The sequencing was conducted using BigDye 3.1 chemistry and ABI3500xl genetic analyzer (Applied Biosystems, Foster City, CA, USA) according to the manufacturer’s protocol.

### Bioinformatics

The SNP proximity search was performed using the SNAP software with genomic data collected by the HapMap Consortium and 1000 Genomes Project. The genotypes of untested SNPs were predicted using the IMPUTE2 software [21] and analyzed with the SNPTESTv2 software (6), which is able to take into account genotype uncertainty during association tests. The search for potential binding sites of transcriptional factors in the promoter region of the *CMA1* gene was performed online using Genomatix with the MatInspector program (http://www.genomatix.de).

### Statistical Analysis

The SAS software (version 9.3; SAS Institute Inc, Cary, NC, USA) was used for the database management and statistical analysis. A stepwise selection method based on the Schwarz Bayesian information criterion was implemented to select a model with clinical covariates. The echocardiographic parameters were not Gaussian distributed and were therefore natural log-transformed before their analysis. The primary quantitative outcome variable of the association analyses was LVM/BSA. The principal explanatory variables were the four genotyped *CMA1* htSNPs and the inferred haplotypes. We tested the departure of each SNP from the Hardy-Weinberg equilibrium using Haploview 4.0 [22]. The SNPs were analyzed under an additive genetic model, and the results were used to identify significant associations and to investigate the most appropriate genetic model for each case. Generalized linear models were used to analyze the effects of multiple covariates on a continuous outcome. Haplotypes were inferred for individuals with ambiguous phase, and the haplotype frequencies were estimated and analyzed using the SimHap software [23]. P-values <0.0125 were designated as significant based on the Bonferroni correction for multiple comparisons of the four htSNPs, which were evaluated as independent statistical tests using the simpleM algorithm [24]. P-values <0.05 were considered suggestively associated. The mean values presented in the text are back-transformed to the original scale from the natural log-transformed data shown in tables.

## Results

### Gender Differences in the Left Ventricle Geometry and Function in Patients with Aortic Stenosis

A total of 648 (274 women, 374 men) patients met all of the criteria for inclusion in the study (Table 1). The women were significantly older (p<0.0001) and had a lower body mass index (BMI) than the male patients (p = 0.0496). The ejection fraction (EF) (p<0.0001), mean aortic gradient (p = 0.0008), and maximal aortic gradient (p = 0.0006) were greater in the women, but the LVM (p<0.0001), LVM indexed to BSA (p<0.0001), height (p<0.0001) or height^2.7^ (p = 0.0104), uncorrected LVEDD (p<0.0001), and LVEDD indexed to height (p = 0.0024) were significantly lower in the women compared with the men. The LVEDD indexed to BSA was similar in the women and men (p = 0.9881). Interestingly, the uncorrected IVST was lower in the women (p = 0.0003). However, after correction for BSA or height, the IVST appeared to be greater in the women than the men (p<0.0001 and p = 0.0446 for correction for BSA and height, respectively), and similar results were observed for PWT. The RWT was significantly higher in the women compared with the men (p = 0.0009). Furthermore, concentric hypertrophy was more frequent in women than in men (p = 0.0002), but the prevalence of eccentric hypertrophy was lower in the women (p = 0.0028).

**Table 1:** Demographic Characteristics and Echocardiographic Data for Patients With Aortic Stenosis.

	Female (N = 274)		Male (N = 374)		
	Mean ± SD	Range	Mean ± SD	Range	p-value
Age, y	63.68±10.63	24 to 82	59.7±10.44	18 to 84	<0.0001
Height, cm	160.46±6.07	140 to 182	172.18±5.83	140 to 188	<0.0001
Weight, kg	69.04±12.74	37 to 120	77.24±11.39	47 to 123	<0.0001
BMI, kg/m^2^	26.82±4.8	14.87 to 44.62	26.06±3.66	16.26 to 41.1	0.0496
EF, %	66.28±11.91	21 to 90	60.06±15.64	8 to 92	<0.0001
Maximal aortic gradient, mm Hg	104.21±27.86	40 to 200	95.87±27.26	25 to 218	0.0006
Mean aortic gradient, mm Hg	64.32±19.63	28 to 130	58.81±17.92	17 to 126	0.0008
LVM, g	327.16±88.93	177.53 to 722.15	419.69±118.12	208.01 to 1027.64	<0.0001
LVM/BSA, g/m^2^	191.4±54.38	104.53 to 452.62	221.79±64.04	106.26 to 514.64	<0.0001
LVM/height, g/m	204.12±56.25	113.54 to 457.06	243.93±69.09	120.14 to 608.38	<0.0001
LVM/height^2.7^, g/m^2.7^	91.85±27.17	50.37 to 210.7	97.27±29.7	43.82 to 343.36	0.0104
LVEDD, mm	48.32±5.98	34 to 71	53.98±7.7	37 to 83	<0.0001
LVEDD/BSA, mm/m^2^	28.3±4.2	19.28 to 47.59	28.56±4.64	19.36 to 49.18	0.9881
LVEDD/height, mm/m	30.14±3.81	21.25 to 45.51	31.37±4.48	21.26 to 49.11	0.0024
IVST, mm	14.02±2.33	9 to 25	14.69±2.5	9 to 25	0.0003
IVST/BSA, mm/m^2^	8.21±1.53	5.1 to 14.8	7.77±1.43	4.89 to 14.5	<0.0001
IVST/height, mm/m	8.75±1.51	5.49 to 15.82	8.55±1.54	5.2 to 17.14	0.0446
PWT, mm	13.23±2.02	8 to 25	13.86±1.98	9 to 21	<0.0001
PWT/BSA, mm/m^2^	7.77±1.42	4.7 to 14.75	7.33±1.16	4.61 to 11.55	<0.0001
PWT/height, mm/m	8.26±1.33	4.91 to 15.82	8.06±1.19	5.2 to 12.07	0.0382
RWT	0.56±0.12	0.3 to 1.19	0.53±0.11	0.25 to 1.03	0.0009
LV geometry, N (%)					
concentric hypertrophy	236 (86.1%)		277 (74.0%)		0.0002
eccentric hypertrophy	38 (13.9%)		87 (23.3%)		0.0028
concentric remodeling	0 (0.0%)		9 (2.4%)		
normal geometry	0 (0.0%)		1 (0.3%)		
Hypertension, N (%)	128 (49%)		123 (34%)		
Diabetes, N (%)	13 (4%)		22 (5%)		
Significant coronary artery disease	46 (18%)		90 (25%)		
NYHA, N (%)					
I	12 (4%)		27 (7%)		
II	57 (22%)		101 (28%)		
III	113 (43%)		133 (37%)		
IV	71 (27%)		83 (23%)		

### Building Multivariable Models with Clinical Covariates

Table S1 in File S1 outlines the clinical covariate associations in the multivariable model of each of the echocardiographic parameter in detail. Age and gender were forced into a model. Moreover, of the variables EF, maximal aortic gradient, NYHA, mean aortic gradient, significant coronary artery disease, and hypertension, the statistical analysis based on the Schwarz Bayesian information criterion selected the variables EF and maximal aortic gradient to represent most of the echocardiographic phenotypes.

### Tagging SNP and Haplotypes

**Figure 1:**
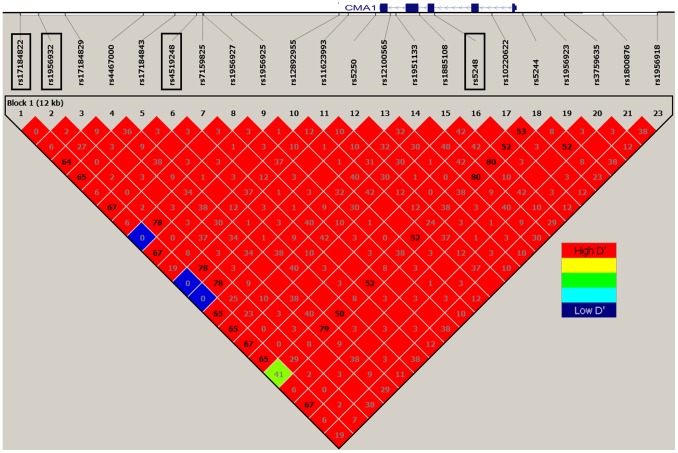
Linkage Disequilibrium structure at the *CMA1* gene locus. Linkage Disequilibrium pattern (Heat Map) is represented by pairwise D′ values between SNPs with MAF>5% based on genotypes from HapMap (CEU population). r2 values (x100) calculated for each SNPs pair are given in the squares. Blank squares represent r2 values equal to 100. Rectangles are used to indicate Tagging SNPs.

We surveyed the genetic variation across a 10.4-kb region spanning the *CMA1* locus: from 2.6 kb upstream of the first exon through 5.1 kb downstream of the transcribed region. One haplotype block encompassing the whole *CMA1* locus was defined (Figure 1), and four htSNPs (rs17184822, rs1956932, rs4519248, and rs5248) were selected to predict the four common (>5%) haplotypes among Caucasians (R_h_^2^>0.90).

### Associations between htSNPs and Left Ventricular Mass Index

**Figure 2:**
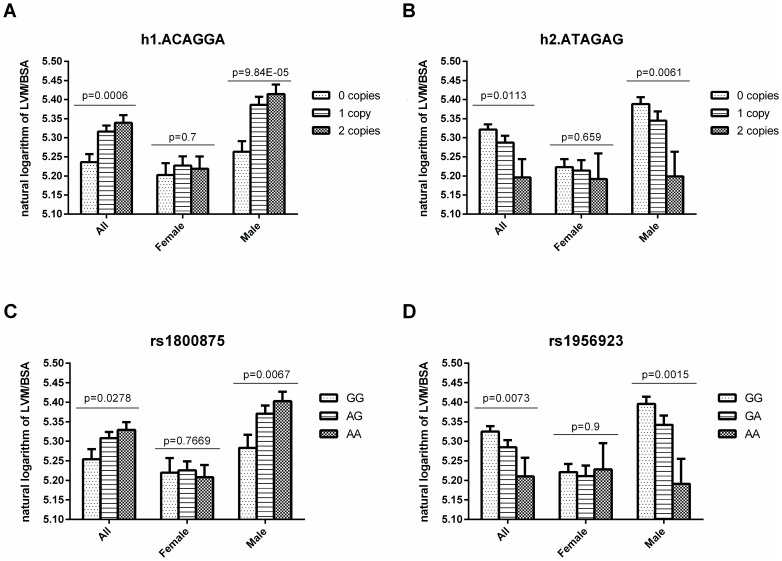
*CMA1* polymorphisms and heart mass in patient with aortic stenosis. Association between haplotypes h1.ACAGGA (A) and h2.ATAGAG (B) in the *CMA1* gene and potentially functional polymorphisms rs1800875 (C) and rs1956923 (D) in the promoter region of *CMA1* gene and natural logarithm of LVM/BSA in patients with aortic stenosis. Values are means with the SEM indicated by error bars.

The single htSNP analyses (Table 2) of all of the patients, which were corrected for multiple comparisons, identified a significant genetic association with low LVM/BSA for the T allele of rs4519248, and an additive genetic model was suggested (mean: 205.6 for CC variant, 197.0 for CT, and 182.9 for TT, p = 0.0053). The association was moderately reduced by the adjustment for age, sex, ejection fraction, and maximal aortic gradient (full model, p = 0.0215). The htSNP rs4519248 is located 3.7 kb from the 3′UTR of the *CMA1* gene and might not be biologically functional but rather a marker of a functional variant. Using a pairwise linkage disequilibrium threshold r2>0.8, we have found two SNPs in the promoter region of the *CMA1* gene: rs1956923 and rs1800876 which are in complete linkage disequilibrium (r2 = 1.0) with rs4519248. The transcription factor binding site (TFBS) analysis revealed that the rs1956923, but not the rs1800876, affects the binding of a few transcription factors to DNA, and this polymorphism was therefore selected for further analysis (Table S2 in File S1). In addition, we have genotyped the rs1800875 polymorphism located in the promoter region, because the rs1800875 is the most frequently analyzed SNP in the CMA1 locus and its associations with certain diseases were found. The genotypic analysis (Table 2 and Figure 2) showed a significant association of the A allele of rs1956923 (p = 0.0073) and a suggestive association of the G allele of rs1800875 (p = 0.0278) with low LVM/BSA in the additive genetic model in all of the patients. The association was suggestive for rs1956923 (p = 0.0389) and disappeared for rs1800875 (p = 0.2775) after adjustment for clinical factors in the full model. The gender-specific analyses revealed that the A allele of rs1956923 was significantly associated (Table 2 and Figure 2) with lower LVM/BSA in the crude (mean: 220.5 for GG variant, 208.9 for GA, and 179.6 for AA, p = 0.0015) and full (p = 0.0057) models in males, and the similar finding was obtained for the T allele of rs4519248 (p = 0.0024 for crude and p = 0.0084 for full model) and the G variant of rs1800875 (mean: 222.1 for AA variant, 215.1 for AG, and 197.0 for GG, p = 0.0067 for crude and p = 0.0421 for full model). No significant SNP associations were found among the female patients.

**Table 2:** Association between tagging and potentially functional SNP in the CMA1 gene and natural logarithm of LVM/BSA.

	Mean (SE) [N]					
	Crude			Adjusted*		
	All	Female	Male	All	Female	Male
Tagging SNPs:						
rs17184822						
GG	5.306 (0.012) [536]	5.219 (0.018) [226]	5.370 (0.016) [310]	5.297 (0.011) [531]	5.219 (0.016) [225]	5.372 (0.014) [306]
GT	5.293 (0.028) [99]	5.225 (0.042) [41]	5.341 (0.036) [58]	5.292 (0.025) [98]	5.230 (0.037) [41]	5.351 (0.033) [57]
TT	5.484 (0.198) [2]	5.534 (0.267) [1]	5.433 (0.273) [1]	5.504 (0.175) [2]	5.518 (0.237) [1]	5.495 (0.252) [1]
p-value	0.8747	0.6261	0.5194	0.8892	0.5336	0.6780
rs1956932						
AA	5.308 (0.012) [503]	5.222 (0.018) [211]	5.370 (0.016) [292]	5.298 (0.011) [498]	5.223 (0.016) [210]	5.372 (0.015) [288]
AG	5.289 (0.025) [125]	5.207 (0.036) [54]	5.352 (0.032) [71]	5.291 (0.022) [124]	5.200 (0.032) [54]	5.365 (0.030) [70]
GG	5.301 (0.077) [13]	5.179 (0.109) [6]	5.406 (0.103) [7]	5.298 (0.068) [13]	5.256 (0.096) [6]	5.365 (0.095) [7]
p-value	0.5634	0.6148	0.8244	0.8391	0.7472	0.8246
rs4519248						
CC	5.326 (0.014) [368]	5.228 (0.022) [149]	5.393 (0.018) [219]	5.312 (0.013) [365]	5.224 (0.019) [149]	5.391 (0.017) [216]
CT	5.283 (0.018) [239]	5.210 (0.026) [104]	5.339 (0.023) [135]	5.282 (0.016) [236]	5.220 (0.023) [103]	5.347 (0.022) [133]
TT	5.209 (0.046) [37]	5.218 (0.061) [19]	5.199 (0.064) [18]	5.232 (0.040) [37]	5.206 (0.054) [19]	5.235 (0.059) [18]
p-value	0.0053#	0.6718	0.0024#	0.0326	0.7752	0.0084#
rs5248						
AA	5.305 (0.012) [556]	5.218 (0.017) [234]	5.368 (0.015) [322]	5.297 (0.011) [550]	5.215 (0.015) [233]	5.374 (0.014) [317]
AG	5.305 (0.031) [79]	5.234 (0.045) [35]	5.362 (0.041) [44]	5.299 (0.028) [79]	5.256 (0.040) [35]	5.351 (0.038) [44]
GG	5.484 (0.197) [2]	5.534 (0.266) [1]	5.433 (0.272) [1]	5.504 (0.175) [2]	5.517 (0.235) [1]	5.494 (0.252) [1]
p-value	0.7730	0.4789	0.9416	0.6928	0.1913	0.6946
Potentially functional SNPs in the promoter of CMA1 gene:						
rs1800875						
AA	5.329 (0.020) [202]	5.208 (0.031) [77]	5.403 (0.024) [125]	5.300 (0.018) [202]	5.204 (0.027) [77]	5.387 (0.022) [125]
AG	5.308 (0.016) [303]	5.226 (0.023) [133]	5.371 (0.021) [170]	5.308 (0.014) [298]	5.229 (0.021) [132]	5.384 (0.019) [166]
GG	5.254 (0.026) [116]	5.220 (0.037) [53]	5.283 (0.034) [63]	5.262 (0.023) [116]	5.225 (0.033) [53]	5.297 (0.031) [63]
p-value	0.0278	0.7669	0.0067#	0.2775	0.5756	0.0421
rs1956923						
GG	5.325 (0.015) [367]	5.221 (0.022) [149]	5.396 (0.018) [218]	5.310 (0.013) [364]	5.218 (0.019) [149]	5.393 (0.017) [215]
GA	5.285 (0.018) [236]	5.211 (0.027) [102]	5.342 (0.023) [134]	5.284 (0.016) [233]	5.222 (0.024) [101]	5.350 (0.022) [132]
AA	5.210 (0.045) [39]	5.228 (0.060) [20]	5.191 (0.062) [19]	5.229 (0.039) [39]	5.207 (0.053) [20]	5.228 (0.058) [19]
p-value	0.0073#	0.9058	0.0015#	0.0389	0.9595	0.0057#

*Adjusted for gender, age, ejection fraction, maximal aortic gradient.

# significance after correction for multiple comparisons: p<0.0125.

### Associations between Haplotypes in the *CMA1* Gene and LVM/BSA

The haplotypes were reconstructed using the six genotyped rs5248, rs4519248, rs1956932, rs17184822, rs1956923, and rs1800875, and the analyses were performed using the additive genetic model. The haplotype h1.ACAGGA (51.46%) was significantly associated with higher LVM/BSA in the total group of patients (Table 3 and Figure 2; p = 0.0006), and this association was suggestive after adjustment for clinical factors (p = 0.0169). The analysis showed a significant association of the h2.ATAGAG (23.09%) haplotype with low LVM/BSA in the crude model (p = 0.0113), and a suggestive association was found in the full model (p = 0.0319). The associations were stronger among male patients (Table 3 and Figure 2): h1.ACAGGA (mean: 193.1 for zero copies, 218.3 for one copy, 224.5 for two copies, p = 9.84×10^−5^ and p = 0.00075 for the crude and full models, respectively), h2.ATAGAG (mean: 218.8 for zero copies, 209.6 for one copy, 181.1 for two copies, p = 0.0061 and p = 0.0168 for the crude and full models, respectively). In contrast, no significant LVM/BSA differences were found in women (Table 3).

**Table 3:** Association between haplotypes in the CMA1 gene and natural logarithm of LVM/BSA.

	Mean (SE)					
	Crude			Adjusted*		
	All	Female	Male	All	Female	Male
h1.ACAGGA	51.46%	49.71%	52.75%	51.46%	49.71%	52.75%
0 copies	5.236 (0.021)	5.202 (0.031)	5.263 (0.028)	5.255 (0.019)	5.218 (0.029)	5.260 (0.026)
1 copy	5.316 (0.016)	5.227 (0.024)	5.386 (0.021)	5.322 (0.014)	5.224 (0.021)	5.384 (0.019)
2 copies	5.339 (0.020)	5.219 (0.032)	5.414 (0.025)	5.320 (0.018)	5.215 (0.029)	5.384 (0.024)
p-value	0.0006#	0.7000	9.84E-05#	0.0169	0.93617	0.00075#
h2.ATAGAG	23.09%	24.43%	22.17%	23.09%	24.43%	22.17%
0 copies	5.321 (0.014)	5.223 (0.021)	5.388 (0.018)	5.317 (0.013)	5.223 (0.020)	5.372 (0.017)
1 copy	5.287 (0.018)	5.214 (0.027)	5.345 (0.024)	5.295 (0.016)	5.226 (0.024)	5.337 (0.022)
2 copies	5.196 (0.048)	5.192 (0.067)	5.199 (0.064)	5.216 (0.042)	5.159 (0.059)	5.220 (0.060)
p-value	0.0113#	0.6590	0.0061#	0.0319	0.55027	0.0168
h3.ACGGGG	11.17%	11.14%	11.11%	11.17%	11.14%	11.11%
0 copies	5.305 (0.012)	5.219 (0.018)	5.369 (0.016)	5.306 (0.011)	5.223 (0.017)	5.357 (0.015)
1 copy	5.284 (0.025)	5.212 (0.037)	5.336 (0.032)	5.294 (0.022)	5.203 (0.034)	5.336 (0.030)
2 copies	5.356 (0.084)	5.247 (0.120)	5.447 (0.111)	5.339 (0.074)	5.299 (0.106)	5.357 (0.103)
p-value	0.7420	0.9100	0.6850	0.842	0.92301	0.6159
h4.GCATGG	5.54%	5.84%	5.30%	5.54%	5.84%	5.30%
0 copies	5.301 (0.012)	5.213 (0.017)	5.365 (0.015)	5.302 (0.010)	5.214 (0.016)	5.353 (0.014)
1 copy	5.304 (0.034)	5.250 (0.049)	5.348 (0.045)	5.312 (0.030)	5.261 (0.044)	5.339 (0.042)
2 copies	5.509 (0.161)	5.535 (0.266)	5.497 (0.193)	5.497 (0.142)	5.518 (0.235)	5.527 (0.177)
p-value	0.5690	0.2830	0.9330	0.424	0.16564	0.8914
h5.rare	–	–	–	–	–	–
0 copies	5.306 (0.012)	5.219 (0.017)	5.371 (0.015)	5.307 (0.010)	5.220 (0.016)	5.360 (0.014)
1 copy	5.325 (0.035)	5.288 (0.058)	5.344 (0.042)	5.309 (0.031)	5.251 (0.053)	5.329 (0.039)
2 copies	5.130 (0.059)	5.103 (0.072)	5.173 (0.094)	5.220 (0.054)	5.186 (0.065)	5.197 (0.093)
p-value	0.0628	0.4430	0.0780	0.266	0.85140	0.1091

*Adjusted for gender, age, ejection fraction, maximal aortic gradient.

# significance after correction for multiple comparisons: p<0.0125.

### Search for the Functional Variant: Sequencing of the *CMA1* Gene and TFBS Analysis

To maximize the probability to detect functional mutations, we selected sixteen males for sequencing, as described in the supplementary materials (Table S3 in File S1), and sequenced 6 kb of the promoter region, introns, and exons and 2 kb downstream of the *CMA1* gene. The sequencing revealed 19 variants in the *CMA1* locus (Table S3 and Table S4 in File S1). We then examined all of the SNPs found in the *CMA1* gene through sequencing with MAF>5% to determine their association with LVM/BSA in males. The genotypes of the untested SNPs were imputed from the genotypes of the htSNPs and reference haplotypes available from the 1000 Genomes Project. The minor allele of rs1956923 showed the most significant protective effect against cardiac hypertrophy (Table S4 in File S1). We then performed a TFBS analysis for each frequent (MAF>5%) SNP found by sequencing and that was significantly associated with LVM/BSA. The results are presented in Table S2 in File S1. Interestingly, the protective allele of rs1956923 disrupts the binding site of the cAMP-responsive element binding protein (CREB), which is known to be one of the primary targets for signaling pathways that induce the transcriptional activation of hypertrophy-associated genes with its co-activators, such as CBP and p300 [25].

### Associations between the rs1956923 Polymorphism in the Promoter Region of the *CMA1* Gene and Echocardiographic Parameters

The genotypic analysis (Table 4) showed a significant association of the A allele of rs1956923 with low LVM in the additive genetic model, regardless of the indexation method used in the crude and full models. Similar results were found for LVEDD. No significant differences in the IVST, IVST/BSA, IVST/height, PWT, PWT/BSA, PWT/height, and RWT were found between the groups of male patients defined by the rs1956923 genotype.

**Table 4:** Mean values of the echocardiographic parameters Based on rs1956923 Genotypes in males.

	Crude				Adjusted*			
	GG (N = 218)	GA (N = 134)	AA (N = 19)	p-value	GG (N = 215)	GA (N = 132)	AA (N = 19)	p-value
log LVM/BSA, g/m^2^	5.396 (0.018)	5.342 (0.023)	5.191 (0.062)	0.0015	5.393 (0.017)	5.350 (0.022)	5.228 (0.058)	0.0057
log LVM/height, g/m	5.489 (0.018)	5.441 (0.023)	5.291 (0.061)	0.0025	5.488 (0.017)	5.448 (0.022)	5.326 (0.057)	0.0082
log LVM/height^2.7^, g/m^2.7^	4.564 (0.019)	4.522 (0.024)	4.368 (0.063)	0.0054	4.563 (0.018)	4.529 (0.023)	4.401 (0.060)	0.0167
log LVEDD, mm	3.994 (0.009)	3.962 (0.012)	3.938 (0.032)	0.0122	3.990 (0.008)	3.972 (0.010)	3.934 (0.026)	0.0222
log LVEDD/BSA, mm/m^2^	3.356 (0.010)	3.322 (0.013)	3.294 (0.035)	0.0141	3.352 (0.009)	3.333 (0.011)	3.293 (0.030)	0.0340
log LVEDD/height, mm/m	3.450 (0.009)	3.421 (0.012)	3.395 (0.032)	0.0186	3.447 (0.008)	3.431 (0.010)	3.390 (0.026)	0.0335
log IVST, mm	2.677 (0.011)	2.678 (0.014)	2.606 (0.038)	0.2897	2.681 (0.010)	2.670 (0.013)	2.638 (0.034)	0.2243
log IVST/BSA, mm/m^2^	2.039 (0.012)	2.038 (0.015)	1.963 (0.040)	0.2397	2.042 (0.011)	2.031 (0.014)	1.997 (0.037)	0.2349
log IVST/height, mm/m	2.133 (0.012)	2.137 (0.015)	2.063 (0.039)	0.3714	2.137 (0.010)	2.129 (0.013)	2.094 (0.035)	0.3113
log PWT, mm	2.623 (0.009)	2.624 (0.012)	2.544 (0.032)	0.1527	2.626 (0.009)	2.618 (0.011)	2.569 (0.029)	0.1228
log PWT/BSA, mm/m^2^	1.986 (0.010)	1.984 (0.013)	1.900 (0.035)	0.1334	1.988 (0.010)	1.979 (0.012)	1.928 (0.033)	0.1491
log PWT/height, mm/m	2.079 (0.010)	2.083 (0.012)	2.000 (0.033)	0.2176	2.082 (0.009)	2.078 (0.011)	2.025 (0.030)	0.1905
log RWT	−0.678 (0.015)	−0.645 (0.019)	−0.701 (0.050)	0.5051	−0.671 (0.012)	−0.661 (0.016)	−0.672 (0.041)	0.7126

*adjusted for age, ejection fraction, maximal aortic gradient,

## Discussion

In this comprehensive study of the chymase gene, we identified a common genetic variation in the *CMA1* gene as an independent predictor of left ventricular mass in male patients with aortic stenosis. The common haplotype h1.ACAGGA, which was inferred by combining six individual htSNPs, is associated with risk for cardiac hypertrophy in the additive model. In contrast, the protective h2.ATAGAG haplotype is associated with low LVM/BSA in the additive model. The haplotypes h1.ACAGGA and h2.ATAGAG can be tagged by the A allele of rs1800875 and by the A allele of rs1956923, respectively. Because both of these htSNPs are localized in the promoter region of the *CMA1* gene, it may be inferred that these polymorphisms affect the expression of *CMA1* gene themselves or are in close linkage with functional variants. Comparative sequencing of the *CMA1* locus in individuals who are homozygous for risk or protective haplotypes revealed additional candidates for functional variants in the promoter region of the gene. The new variants were tested for their association with LVM/BSA, and rs1956923 exhibited the strongest effect on the left ventricular mass. Moreover, we conducted a test conditioning upon the rs1956923 to search for secondary signals of association and found no significant results. The TFBS analysis of the new variants indicated that rs1956923 was the most likely functional polymorphism, and its minor allele A disrupts the binding site of the transcription factor CREB. Phosphorylated CREB can recruit the CBP co-activator and initiate the cAMP-dependent activation of gene expression [26]. It has been shown that CBP and the closely related p300 protein play an important role in the process of cardiac hypertrophy [25].

We found an association between the genetic variation in the *CMA1* gene and the degree of LVH only in men. This phenomenon can be explained by the fact that gender has a profound impact on the cardiac remodeling response to pressure overload in patients with aortic stenosis. In our study, women had a greater relative wall thickness and better systolic function, as determined by the ejection fraction, and these differences have been reported by other researchers [27], [28]. In our cohort, women had higher mean and maximal transvalvular gradients, and similar trends were observed in other studies [27], [28], [29] with a small number of patients. Compared with men, the women had significantly lower LVM indexed to BSA, height or height^2.7^. These results are in agreement with two previous studies [29], [30] but contrary to two other studies, which showed that gender differences in LVM/BSA were not statistically significant, although a trend toward a greater LVM/BSA in women was observed [27], [28]. Moreover, the uncorrected IVST and PWD were significantly lower in women, and similar differences, albeit without significance, were shown in a previous study [29], but opposite results were found by other studies [27], [28]. After indexation to BSA or height, the IVST and PWD were significantly greater in women, and similar differences were previously reported [27], [28]. In absolute terms, the LVDD was greater in men, as found in previous studies [27], [28], [29], [30]. However, after indexation to BSA, this difference disappeared in our as well other studies [29], although one study showed a trend toward higher LVDD/BSA values in women compared with men [28]. In a healthy population, the LVDD/BSA values [31] and their normal range [32] are higher in women than in men. Thus, the lack of a gender-related difference in LVDD/BSA in aortic stenosis patients found in our study may be treated as a greater enlargement of the LV cavity in men compared with women. Our results on the gender-related difference in LVDD indexed to height appear to support this hypothesis. The small sample size, older age, and lower mean transvalvular gradient in two previous studies [28], [29] and the small number of patients and the younger population included in a third previous study [27] may account for the discrepancies between our results and those obtained in these previous studies. With the use of properly indexed LV dimensions and a large number of patients, our study showed that men with severe aortic stenosis develop a more eccentric form of hypertrophy than women, and this more eccentric form is characterized by thinner LV walls, more ventricular dilatation, lower transvalvular gradients, smaller relative wall thickness, and worse systolic function.

The above-described sex-related differences in the echocardiographic parameters have representations at the cellular level because gender-specific pathways in the cardiac response to pressure overload have been found in animal models [33], and the role of estrogens have been shown [34], [35].

In a mouse model of pressure overload hypertrophy, males exhibited more severe tissue fibrosis, LV dilation, and hemodynamic dysfunction, as well as the upregulation of TGF-β genes and TGF-β target genes, such as collagens and fibronectin. Orchidectomy reduced both the specific mRNA content and the fibrosis and hemodynamic dysfunction. The exposure of fibroblasts or cardiomyocytes to physiological concentrations of dihydrotestosterone significantly increased the mRNA levels of TGF-β [36]. This result suggests that TGF-β is a downstream effector of androgens and plays a crucial role in LVH, fibrosis, dilatation, and dysfunction. TGF-β may be responsible for the gender-related differences in cardiac remodeling between postmenopausal women, who are no longer under the influence of estrogens, and older men, many of whom still have circulating testosterone concentrations close to those of young men [28], [29], [37]. Animal and experimental models agree with the clinical studies that have been conducted on aortic stenosis patients, which showed more pronounced interstitial fibrosis [37] and greater upregulation of matrix-related genes in male hearts [38].

We would like to emphasize that experimental [36] and clinical [39] data demonstrate that the TGF-β pathway containing Smad2 is upregulated and affects cardiac fibrosis and eccentric remodeling only in males with chronic pressure overload. Therefore, changes in the availability of the active form of TGF-β can influence the LV mass and LVDD in male patients with aortic stenosis only. Chymase contributes to the activation of TGF-β [10]; thus, the functional polymorphism affecting the expression of the chymase gene may influence the activation of TGF-β and thereby modify the cardiac remodeling response to pressure overload in men. Therefore, in our study, we found an association between the rs1956923 polymorphism in the promoter region of the *CMA1* gene and the LV mass and LVDD but not IVST, PWD, and RWT in the group of male patients only.

To date, only a few studies have evaluated the impact of genetic polymorphisms on LVH in patients with aortic stenosis [4], [40], [41], and all of these studies had too-small sample sizes (from 54 to 105 patients) and were thus unable to minimize both false positive and false negative errors, stratify for gender, and test for truth associations. Interestingly, one study showed a minor trend of the G allele of rs1800875 toward a lower LVM/BSA in an additive genetic model in all patients, but the difference did not reach statistical significance (p = 0.27). Our study size and design allowed us to address the weaknesses of the former studies. The htSNPs were selected to maximally account for the genetic variation in the *CMA1* gene and to perform single SNP and complementary haplotype analysis to decrease the false negative errors.

### Implications of Study Findings

The results of our study may be applied to individual risk prediction and improvement in the timing of valve replacement. In addition, the results may stimulate the implementation of new therapeutic interventions based on the administration of chymase inhibitors to patients bearing the risk haplotype.

### Study Limitations

Functional studies are required to elucidate the details of the molecular mechanism through which the functional SNP or haplotype affects the *CMA1* promoter activity in cardiac cells subjected to pressure overload. In addition, the present study consisted of Caucasians, and the results may not apply to other populations.

## Conclusions

Common genetic variants and haplotypes at the promoter of the *CMA1* gene are associated with LVH in male patients with aortic stenosis. The gender-related differences in the cardiac remodeling response to pressure overload are substantial if properly indexed heart dimensions are used, and these differences impose the search for gender-specific pathways. Although a causal molecular mechanism has not been established, the results of our study can be used for the risk stratification of men with aortic stenosis and for pharmacogenetics-guided therapy in the future.

## Supporting Information

File S1 **Table S1,** Multivariable modeling of clinical covariates with echocardiographic parameters. **Table S2,** Transcription Factor Binding Site analysis of SNPs found in the *CMA1* locus by sequencing. **Table S3,** Variants found in the *CMA1* locus by sequencing. **Table S4,** Association between SNPs found in *CMA1* locus by sequencing and natural logarithm of Left Ventricular Mass Index in males.
(DOCX)
