# Supplementary material for: Association of the Common Genetic Polymorphisms and Haplotypes of the Chymase Gene with Left Ventricular Mass in Male Patients with Symptomatic Aortic Stenosis
Source: PLoS One. 2014 May 13;9(5):e96306. doi: 10.1371/journal.pone.0096306 (PMC4019480; doi:10.1371/journal.pone.0096306)
Supplement: File S1 — Table S1, Multivariable modeling of clinical covariates with echocardiographic parameters. Table S2, Transcription Factor Binding Site analysis of SNPs found in the CMA1 locus by sequencing. Table S3, Variants found in the CMA1 locus by sequencing. Table S4, Association between SNPs found in CMA1 locus by sequencing and natural logarithm of Left Ventricular Mass Index in males. (DOCX) [file pone.0096306.s001.docx]

Table S1. Multivariable modeling of clinical covariates with echocardiographic parameters.

| Dependent variable | Effect Entered | Model R^2^ | Adjusted R^2^ | p-value | Selected* |
| --- | --- | --- | --- | --- | --- |
|  |  |  |  |  |  |
| log LVM/BSA, g/m^2^ | Age, y | 0.000 | -0.002 | 0.7247 | + |
|  | Male gender | 0.062 | 0.058 | <.0001 | + |
|  | EF, % | 0.165 | 0.160 | <.0001 | + |
|  | Maximal aortic gradient, mm Hg | 0.230 | 0.224 | <.0001 | + |
|  | NYHA | 0.252 | 0.240 | 0.0055 |  |
|  | Mean aortic gradient, mm Hg | 0.253 | 0.239 | 0.4772 |  |
|  | Significant coronary artery disease | 0.254 | 0.237 | 0.6702 |  |
|  | Hypertension | 0.254 | 0.236 | 0.8701 |  |
|  |  |  |  |  |  |
| log LVM/height, g/m | Age, y | 0.000 | -0.002 | 0.7131 | + |
|  | Male gender | 0.091 | 0.088 | <.0001 | + |
|  | EF, % | 0.179 | 0.174 | <.0001 | + |
|  | Maximal aortic gradient, mm Hg | 0.238 | 0.232 | <.0001 | + |
|  | NYHA | 0.254 | 0.242 | 0.0313 |  |
|  | Mean aortic gradient, mm Hg | 0.255 | 0.241 | 0.6667 |  |
|  | Significant coronary artery disease | 0.257 | 0.240 | 0.4504 |  |
|  | Hypertension | 0.260 | 0.242 | 0.1876 |  |
|  |  |  |  |  |  |
| log LVM/height^2.7^, g/m^2.7^ | Age, y | 0.002 | 0.000 | 0.3103 | + |
|  | Male gender | 0.010 | 0.006 | 0.0438 | + |
|  | EF, % | 0.107 | 0.102 | <.0001 | + |
|  | Maximal aortic gradient, mm Hg | 0.167 | 0.161 | <.0001 | + |
|  | NYHA | 0.187 | 0.174 | 0.0179 |  |
|  | Mean aortic gradient, mm Hg | 0.188 | 0.173 | 0.465 |  |
|  | Significant coronary artery disease | 0.190 | 0.171 | 0.646 |  |
|  | Hypertension | 0.192 | 0.172 | 0.2353 |  |
|  |  |  |  |  |  |
| log LVEDD, mm | Age, y | 0.010 | 0.008 | 0.0229 | + |
|  | Male gender | 0.141 | 0.137 | <.0001 | + |
|  | EF, % | 0.391 | 0.387 | <.0001 | + |
|  | Maximal aortic gradient, mm Hg | 0.403 | 0.398 | 0.0014 | + |
|  | NYHA | 0.407 | 0.398 | 0.4504 |  |
|  | Mean aortic gradient, mm Hg | 0.407 | 0.397 | 0.7714 |  |
|  | Significant coronary artery disease | 0.408 | 0.395 | 0.7062 |  |
|  | Hypertension | 0.408 | 0.394 | 0.8781 |  |
|  |  |  |  |  |  |
| log LVEDD/BSA, mm/m^2^ | Age, y | 0.001 | -0.001 | 0.5127 | + |
|  | Male gender | 0.001 | -0.003 | 0.6715 | + |
|  | EF, % | 0.297 | 0.293 | <.0001 | + |
|  | Maximal aortic gradient, mm Hg | 0.304 | 0.299 | 0.022 | + |
|  | NYHA | 0.318 | 0.307 | 0.0425 |  |
|  | Mean aortic gradient, mm Hg | 0.320 | 0.307 | 0.2399 |  |
|  | Significant coronary artery disease | 0.320 | 0.305 | 0.8748 |  |
|  | Hypertension | 0.331 | 0.315 | 0.0051 | + |
|  |  |  |  |  |  |
| log LVEDD/height, mm/m | Age, y | 0.001 | -0.001 | 0.4462 | + |
|  | Male gender | 0.018 | 0.014 | 0.0032 | + |
|  | EF, % | 0.325 | 0.321 | <.0001 | + |
|  | Maximal aortic gradient, mm Hg | 0.339 | 0.334 | 0.0014 | + |
|  | NYHA | 0.344 | 0.334 | 0.4047 |  |
|  | Mean aortic gradient, mm Hg | 0.345 | 0.333 | 0.4698 |  |
|  | Significant coronary artery disease | 0.345 | 0.331 | 0.875 |  |
|  | Hypertension | 0.345 | 0.329 | 0.9541 |  |
|  |  |  |  |  |  |
| log IVST, mm | Age, y | 0.002 | 0.000 | 0.3753 | + |
|  | Male gender | 0.019 | 0.015 | 0.0033 | + |
|  | EF, % | 0.064 | 0.058 | <.0001 | + |
|  | Maximal aortic gradient, mm Hg | 0.221 | 0.215 | <.0001 | + |
|  | NYHA | 0.227 | 0.214 | 0.4621 |  |
|  | Mean aortic gradient, mm Hg | 0.227 | 0.213 | 0.4899 |  |
|  | Significant coronary artery disease | 0.230 | 0.213 | 0.4716 |  |
|  | Hypertension | 0.234 | 0.215 | 0.1012 |  |
|  |  |  |  |  |  |
| log IVST/BSA, mm/m^2^ | Age, y | 0.009 | 0.007 | 0.037 | + |
|  | Male gender | 0.034 | 0.030 | 0.0003 | + |
|  | EF, % | 0.053 | 0.047 | 0.0016 |  |
|  | Maximal aortic gradient, mm Hg | 0.193 | 0.187 | <.0001 | + |
|  | NYHA | 0.204 | 0.191 | 0.1514 |  |
|  | Mean aortic gradient, mm Hg | 0.204 | 0.190 | 0.9203 |  |
|  | Significant coronary artery disease | 0.206 | 0.188 | 0.6274 |  |
|  | Hypertension | 0.207 | 0.187 | 0.407 |  |
|  |  |  |  |  |  |
| log IVST/height, mm/m | Age, y | 0.010 | 0.008 | 0.0278 | + |
|  | Male gender | 0.016 | 0.012 | 0.072 | + |
|  | EF, % | 0.052 | 0.046 | <.0001 | + |
|  | Maximal aortic gradient, mm Hg | 0.199 | 0.192 | <.0001 | + |
|  | NYHA | 0.207 | 0.194 | 0.2905 |  |
|  | Mean aortic gradient, mm Hg | 0.207 | 0.192 | 0.7321 |  |
|  | Significant coronary artery disease | 0.209 | 0.191 | 0.5679 |  |
|  | Hypertension | 0.212 | 0.193 | 0.1381 |  |
|  |  |  |  |  |  |
| log PWT, mm | Age, y | 0.002 | 0.000 | 0.3202 | + |
|  | Male gender | 0.027 | 0.024 | 0.0003 | + |
|  | EF, % | 0.049 | 0.043 | 0.0008 |  |
|  | Maximal aortic gradient, mm Hg | 0.229 | 0.222 | <.0001 | + |
|  | NYHA | 0.252 | 0.240 | 0.0046 |  |
|  | Mean aortic gradient, mm Hg | 0.253 | 0.240 | 0.3055 |  |
|  | Significant coronary artery disease | 0.256 | 0.239 | 0.4198 |  |
|  | Hypertension | 0.259 | 0.241 | 0.1522 |  |
|  |  |  |  |  |  |
| log PWT/BSA, mm/m^2^ | Age, y | 0.010 | 0.008 | 0.0258 | + |
|  | Male gender | 0.035 | 0.031 | 0.0004 | + |
|  | EF, % | 0.039 | 0.034 | 0.1206 |  |
|  | Maximal aortic gradient, mm Hg | 0.186 | 0.180 | <.0001 | + |
|  | NYHA | 0.217 | 0.204 | 0.0008 |  |
|  | Mean aortic gradient, mm Hg | 0.221 | 0.207 | 0.103 |  |
|  | Significant coronary artery disease | 0.221 | 0.203 | 0.9676 |  |
|  | Hypertension | 0.223 | 0.204 | 0.2071 |  |
|  |  |  |  |  |  |
| log PWT/height, mm/m | Age, y | 0.012 | 0.010 | 0.0134 | + |
|  | Male gender | 0.018 | 0.014 | 0.0819 | + |
|  | EF, % | 0.033 | 0.028 | 0.0051 |  |
|  | Maximal aortic gradient, mm Hg | 0.202 | 0.196 | <.0001 | + |
|  | NYHA | 0.229 | 0.216 | 0.0019 |  |
|  | Mean aortic gradient, mm Hg | 0.232 | 0.218 | 0.1833 |  |
|  | Significant coronary artery disease | 0.233 | 0.216 | 0.6437 |  |
|  | Hypertension | 0.236 | 0.217 | 0.2017 |  |
|  |  |  |  |  |  |
| log RWT | Age, y | 0.009 | 0.007 | 0.0301 | + |
|  | Male gender | 0.027 | 0.024 | 0.0024 | + |
|  | EF, % | 0.211 | 0.207 | <.0001 | + |
|  | Maximal aortic gradient, mm Hg | 0.337 | 0.331 | <.0001 | + |
|  | NYHA | 0.346 | 0.336 | 0.1235 |  |
|  | Mean aortic gradient, mm Hg | 0.347 | 0.335 | 0.5887 |  |
|  | Significant coronary artery disease | 0.347 | 0.333 | 0.8447 |  |
|  | Hypertension | 0.348 | 0.332 | 0.3614 |  |

*clinical covariates selected in the final models using stepwise selection method based on the Schwarz Bayesian information criterion

Table S2. Transcription Factor Binding Site analysis of SNPs found in the CMA1 locus by sequencing.

| Variant | Allele | TFBS | TF | Transcription Factor name |
| --- | --- | --- | --- | --- |
| rs1956923 | G->A | lost | ATF6 | Activating transcription factor 6 |
|  | G->A | lost | CREB | cAMP-responsive element binding protein |
|  | G->A | new | FOXH1 | Forkhead box H1 |
|  | G->A | lost | BHLHB2 | Basic helix-loop-helix domain containing, class B2 |
|  | G->A | lost | P53 | Tumor suppressor p53 |
| rs1800876 | G->A | new | NKX-3.1 | Prostate-specific homeodomain protein NKX3.1 |
| rs1800875 | C->T | lost | TAL1-E2A | Complex of Lmo2 bound to Tal-1, E2A proteins |
|  | C->T | new | ISGF3G (IRF9) | Interferon-stimulated transcription factor 3 gamma |
| rs1956920 | A->G | lost | EVI1 | Ecotropic viral integration site 1 encoded factor |
| rs1956919 | T->C | lost | FHXA FHXB | Fork head homologous X |
|  | T->C | lost | VMYB | v-Myb |
|  | T->C | new | RFXDC2 (RFX7) | Regulatory factor X domain containing 2 |
| rs1956918 | G->A | new | CRX | Cone-rod homeobox-containing transcription factor |
|  | G->A | new | BRN5 (CNS-1) | Brn-5, POU-VI protein class |
|  | G->A | new | ACAAT | Avian C-type LTR CCAAT box |
|  | G->A | new | PHOX2 (ARIX) | Phox2a and Phox2b |
|  | G->A | new | EVX1 | Even-skipped homeobox 1 |
|  | G->A | new | MEIS1B-HOXA9 | Meis1b and Hoxa9 complex |
| rs1956917 | A->G | new | ETV3 | Ets variant 3 |
|  | A->G | lost | HSF2 | Heat shock factor 2 |
|  | A->G | lost | NFAT5 | Nuclear factor of activated T-cells 5 |
| rs761988 | T->C | new | STAT6 | Signal transducer and activator of transcription 6 |
| rs56267023 | C->T | new | MYT1L | Myelin transcription factor 1-like |

TFBS - Transcription Factor Binding Site; TF - Transcription Factor abbreviation.

Table S3. Variants found in the CMA1 locus by sequencing.

| Haplotype | LVMI | No. | rs11623993 | rs116914716 | rs5246* | chr14:24977251 | rs139719573 | rs1956923 | rs1800876 | rs1800875 | rs1956920 | rs149378382 | rs1956919 | rs1956918 | rs1956917 | rs761988 | rs56267023 | chr14:24981244 | chr14:24981266:I | rs28656464 | rs79339358 |
| --- | --- | --- | --- | --- | --- | --- | --- | --- | --- | --- | --- | --- | --- | --- | --- | --- | --- | --- | --- | --- | --- |
| h2.GAGATA | low | 1 | CC | GG | GG | GG | GG | AA | AA | CC | GG | CC | TT | GG | AA | CC | CC | GG | CC | GG | TT |
|  |  | 2 | CC | GG | CC | GG | GG | AA | AA | CC | GG | CC | TT | GG | AA | CC | CC | GG | -- | GG | TT |
|  |  | 3 | CC | GG | CC | GG | GG | AA | AA | CC | GG | CC | TT | GG | AA | CC | CC | GG | CC | GG | TT |
|  |  | 4 | CC | GG | CG | GG | GG | AA | AA | CC | GG | CC | TT | GG | AA | CC | CC | GG | -- | GG | TT |
|  | high | 5 | CC | GG | CC | GG | GG | AA | AA | CC | GG | CC | TT | GG | AA | CC | CC | GG | -- | GG | TT |
|  |  | 6 | CC | GG | CC | GG | GG | AA | AA | CC | GG | CC | TT | GG | AA | CC | CC | GG | -- | GG | TT |
|  |  | 7 | CC | GG | CC | GG | GG | AA | AA | CC | GG | CC | TT | GG | AA | CC | CC | GG | -- | GG | TT |
|  |  | 8 | CC | GT | CC | GG | GG | AA | AA | CC | GG | CC | TT | GG | AA | CC | CC | AG | CC | GG | TT |
| h1.AGGACA | low | 9 | TT | GG | CC | GG | GG | GG | GG | TT | AA | CC | CC | AA | GG | TT | TT | GG | -- | AA | -- |
|  |  | 10 | TT | GG | CC | GG | GG | GG | GG | TT | AA | CC | CC | AA | GG | TT | TT | GG | -- | AA | -- |
|  |  | 11 | TT | GG | CC | GG | GG | GG | GG | TT | AA | CC | CC | AA | GG | TT | TT | GG | -- | AA | -- |
|  |  | 12 | TT | GG | CC | GG | GG | GG | GG | TT | AA | CT | CC | AA | GG | TT | TT | GG | -- | AG | -- |
|  | high | 13 | TT | GG | CC | GG | GG | GG | GG | TT | AA | CC | CC | AA | GG | TT | TT | GG | -- | AA | -- |
|  |  | 14 | TT | GG | CC | GG | GG | GG | GG | TT | AA | CC | CC | AA | GG | TT | TT | GG | CC | AA | -- |
|  |  | 15 | TT | GG | CC | GG | AG | GG | GG | TT | AA | CC | CC | AA | GG | TT | TT | GG | -- | AA | -- |
|  |  | 16 | TT | GG | CC | GT | GG | GG | GG | TT | AA | CC | CC | AA | GG | TT | TT | GG | CC | GG | -- |

To maximize the probability to detect functional mutations, we selected sixteen males for the sequencing analyses. Individuals 1 through 4 were homozygous for the h2.ATAGAG haplotype and had low LVM/BSA, and patients 5 through 8 were homozygous for the h2.ATAGAG haplotype and had high LVM/BSA. Similarly, individuals 9 through 16 were homozygous for the h1.ACAGGA haplotype; however, patients 9 through 12 had low LVM/BSA, and patients 13 – 16 had high LVM/BSA. The haplotype h1.ACAGGA, which is associated with the risk for cardiac hypertrophy, is tagged by the A allele of rs1800875. Thus, we expected to find the risk allele of the casual polymorphism only in individuals 9 through 16 if its MAF was similar to the rs1800875 frequency or particularly accumulated in individuals with high LVM/BSA (i.e., patients 13 through 16). The A allele of rs1956923 can be used to tag the protective h2.ATAGAG haplotype; thus, the protective allele of the functional variant should be present only in individuals 1 through 8 if its MAF is similar to the rs1956923 frequency or should cluster in individuals 1 through 4 if its MAF is lower. *Interestingly, rs5246 was clustered in patients with low LVM/BSA and the protective haplotype h2.GAGATA and is responsible for an amino acid change (Gly to Arg) at codon 46 of the chymase protein; however, this SNP has an MAF of 0.007 in the European population according to the 1000Genomes database. We found three variants that were not previously reported in the dbSNP and 1000Genomes databases. The minor alleles of chr14:24977251 and chr14:24981244 were found only once in the 32 sequenced chromosomes. Thus, these two mutations were treated as rare variants. Although chr14:24981266:I was more frequent, both alleles were present on protective and risk haplotypes. Therefore, all three new variants were excluded from further analyses.

Table S4. Association between SNPs found in CMA1 locus by sequencing and natural logarithm of Left Ventricular Mass Index in males.

|  |  | Allele | |  | Crude | | Adjusted* | |
| --- | --- | --- | --- | --- | --- | --- | --- | --- |
|  | Distance^†^ | Major | Minor | MAF | p-value | coeff. | p-value | coeff. |
| rs11623993^‡^ | 3402 | T | C | 0.456 | 0.005 | -0.21 | 0.015 | -0.17 |
| rs116914716 | 3338 | G | T | 0.003 | NA | NA | NA | NA |
| rs5248 | 1657 | T | C | 0.072 | 0.239 | -0.17 | 0.418 | -0.11 |
| rs5246 | 836 | C | G | 0.003 | NA | NA | NA | NA |
| chr14:24977251 | 220 | G | T | NA | NA | NA | NA | NA |
| rs139719573 | -383 | G | A | 0.006 | NA | NA | NA | NA |
| rs1956923 | -496 | G | A | 0.234 | 0.001 | -0.28 | 0.004 | -0.23 |
| rs1800876‡ | -1742 | G | A | 0.223 | 0.002 | -0.28 | 0.007 | -0.22 |
| rs1800875 | -1867 | A | G | 0.416 | 0.006 | -0.20 | 0.033 | -0.15 |
| rs1956920‡ | -1956 | A | G | 0.274 | 0.002 | -0.25 | 0.007 | -0.21 |
| rs149378382 | -2569 | C | T | 0.018 | NA | NA | NA | NA |
| rs1956919‡ | -2822 | C | T | 0.423 | 0.004 | -0.21 | 0.018 | -0.16 |
| rs1956918^‡^ | -2840 | A | G | 0.439 | 0.005 | -0.21 | 0.019 | -0.16 |
| rs1956917^‡^ | -3021 | G | A | 0.300 | 0.005 | -0.23 | 0.018 | -0.18 |
| rs761988^‡^ | -3214 | T | C | 0.226 | 0.002 | -0.29 | 0.006 | -0.23 |
| rs56267023^‡^ | -3565 | T | C | 0.444 | 0.005 | -0.22 | 0.019 | -0.16 |
| chr14:24981244 | -3773 | G | A | NA | NA | NA | NA | NA |
| chr14:24981266:I | -3795 | - | C | NA | NA | NA | NA | NA |
| rs28656464^‡^ | -4369 | A | G | 0.468 | 0.006 | -0.22 | 0.022 | -0.17 |
| rs79339358^‡^ | -5343 | - | T | 0.267 | 0.003 | -0.26 | 0.009 | -0.21 |

*Adjusted for age, ejection fraction, maximal aortic gradient. ^†^distance from transcription start site. ^‡^SNP which genotypes were imputed in a set of study individuals using a set of reference haplotypes form 1000Genomes database and genotypes examined in the study. Rare SNPs with MAF<5% in the European population were removed from the subsequent analyses.
